# Nanoscale Strain-Hardening of Keratin Fibres

**DOI:** 10.1371/journal.pone.0041814

**Published:** 2012-07-25

**Authors:** Patrick Fortier, Sandy Suei, Laurent Kreplak

**Affiliations:** Department of Physics and Atmospheric Science, Dalhousie University, Halifax, Canada; Massachusetts Institute of Technology, United States of America

## Abstract

Mammalian appendages such as hair, quill and wool have a unique structure composed of a cuticle, a cortex and a medulla. The cortex, responsible for the mechanical properties of the fibers, is an assemblage of spindle-shaped keratinized cells bound together by a lipid/protein sandwich called the cell membrane complex. Each cell is itself an assembly of macrofibrils around 300 nm in diameter that are paracrystalline arrays of keratin intermediate filaments embedded in a sulfur-rich protein matrix. Each macrofibril is also attached to its neighbors by a cell membrane complex. In this study, we combined atomic force microscopy based nano-indentation with peak-force imaging to study the nanomechanical properties of macrofibrils perpendicular to their axis. For indentation depths in the 200 to 500 nm range we observed a decrease of the dynamic elastic modulus at 1 Hz with increasing depth. This yielded an estimate of 1.6GPa for the lateral modulus at 1 Hz of porcupine quill’s macrofibrils. Using the same data we also estimated the dynamic elastic modulus at 1 Hz of the cell membrane complex surrounding each macrofibril, i.e., 13GPa. A similar estimate was obtained independently through elastic maps of the macrofibrils surface obtained in peak-force mode at 1 kHz. Furthermore, the macrofibrillar texture of the cortical cells was clearly identified on the elasticity maps, with the boundaries between macrofibrils being 40–50% stiffer than the macrofibrils themselves. Elasticity maps after indentation also revealed a local increase in dynamic elastic modulus over time indicative of a relaxation induced strain hardening that could be explained in term of a α-helix to β-sheet transition within the macrofibrils.

## Introduction

**Figure 1:**
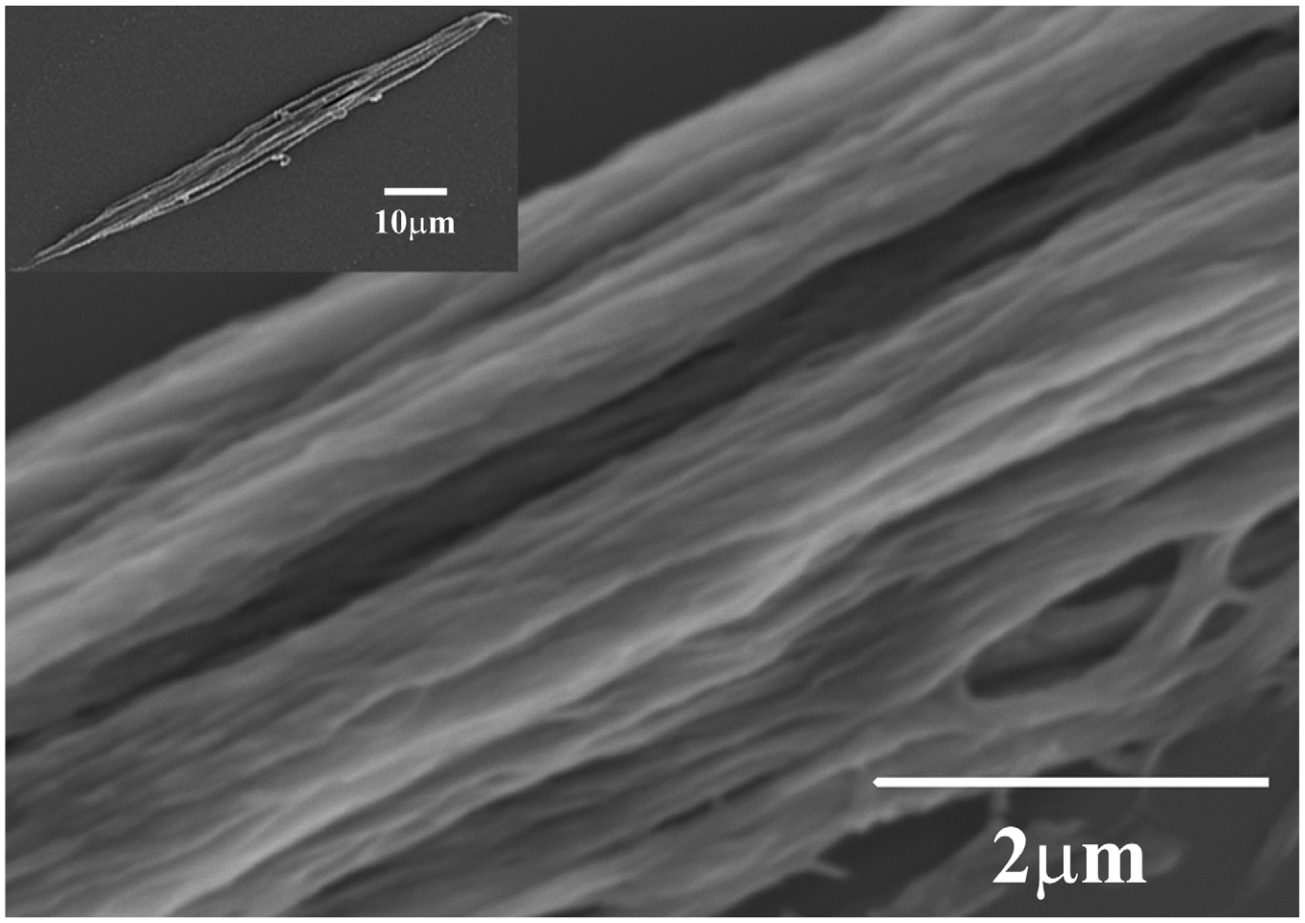
Ultrastructure of a cortical cell. SEM image of cortical cell extracted from a human hair fibre showing the spindle-shape of the cell (inset) as well as the array of macrofibrils.

One of the new avenues in material science is the development of nanocomposites with novel mechanical properties such as dynamic strain hardening [1]. One source of inspiration is Nature’s hierarchical materials such as woods, hooves, beaks and skin appendages. All these materials are nanocomposites, lightweight and exhibit non-linear mechanical properties that are still poorly understood from a structural standpoint. In particular mammalian appendages such as wool, hair and quills have been studied for more than a century. These fibres are composed of three different regions, the cuticule, the cortex and the medulla [2]. The cortex is responsible for the bulk mechanical properties of these fibers [3]. It is a hierarchical fibrous composite from the nanometer to the micrometer scale [2]. The building blocks of the cortex are trichocyte keratins intermediate filaments (IFs) with a 7.5 nm diameter embedded in a sulfur-rich protein matrix and arranged in a paracrystalline hexagonal lattice [4]. The keratin IFs are roughly aligned with the macroscopic fiber axis and arranged into macrofibrils typically 300 nm in diameter [5] (Fig. 1). The macrofibrils are surrounded by a lipid membrane and glued together by an intermacrofibrillar matrix [6], this structure is also named the cell membrane complex (CMC). The macrofibrils are contained in spindle-shaped cortical cells, 100 µm long and several microns wide (Fig. 1, inset).

The macroscopic tensile properties of the cortex have been studied extensively. Below 2% strain the cortex can be considered elastic with a Young’s modulus in the GPa range, beyond 2% strain a yield region is observed until 30% strain followed by a strain-hardening region up to a maximum extensibility of 50 to 100% [7]. The shape and extent of the yield and strain hardening regions are humidity and temperature dependent. At the molecular level, X-ray diffraction and Raman spectroscopy studies revealed that beyond 2–4% strain, the α-helical motif characteristic of the keratin molecules unfolds to form β-sheet structures [8]–[10]. The α-helix to β-sheet transition is considered the molecular explanation for the observed strain-hardening properties of trichocyte keratin fibers [11]. However this process has never been observed at the nanoscale. One way to achieve this goal is to use nano-indentation to apply strains above 30% perpendicular to the macrofibrils’ axis. Typical indenter’s tips are either spherical or conical and pyramidal with large cone or dihedral angles of 100° or more. These indenters provide applied strains of a few percent for the achievable range of indentation depth [12]. However by decreasing the angle, it is possible to achieve applied strains in the order of 30% or more [12]. In this study, we combined standard nano-indentation with a sharp conical tip and peak-force nanomechanical mapping to demonstrate a relaxation induced nanoscale strain-hardening of keratin macrofibrils.

## Materials and Methods

### Samples

Human hairs were a donation of Patrick Fortier. Quills from North American porcupine (*Erethizon dorsatum*) were a gift from Prof. Harm Rotermund.

### Cortical Cell Extraction

Untreated human hairs were lightly washed in deionized (DI) water prior to extraction with a solution of papain-bisulfite (3.3 mg/ml papain (Sigma-Aldrich) in 1% sodium sulfite (Sigma-Aldrich) pH 6.4). Samples were incubated in a water bath at 65°C for 16 hours and stored at 4°C. The digestion left cortical cells and cuticle cells in the solution as well as hair fibers with an exposed cortex. Samples were then centrifuged at 20,000 g for 30 min, pellets were re-suspended, and rinsed in DI water twice to remove salt and cuticle cells prior to imaging. Aliquots were then placed on a glass slide (VWR) and nitrogen dried prior to AFM imaging. The porcupine quills were treated with the same protocol mentioned above apart from the following modifications. Quills were longitudinally dissected using a scalpel blade and rolled open in order to remove the medulla. The cortical part of the quills was then cut into several millimeters squared pieces to facilitate cortical cell extraction.

### Scanning Electron Microscopy (SEM)

Cortical cells extracted from human hairs and resuspended in DI water as described above were placed on a glass slide and sputter coated with palladium and gold for 30 sec (Quorum Technologies, Polaron SC7620) and observed with a Hitachi (S-3000N) SEM at 5 kV.

**Figure 2:**
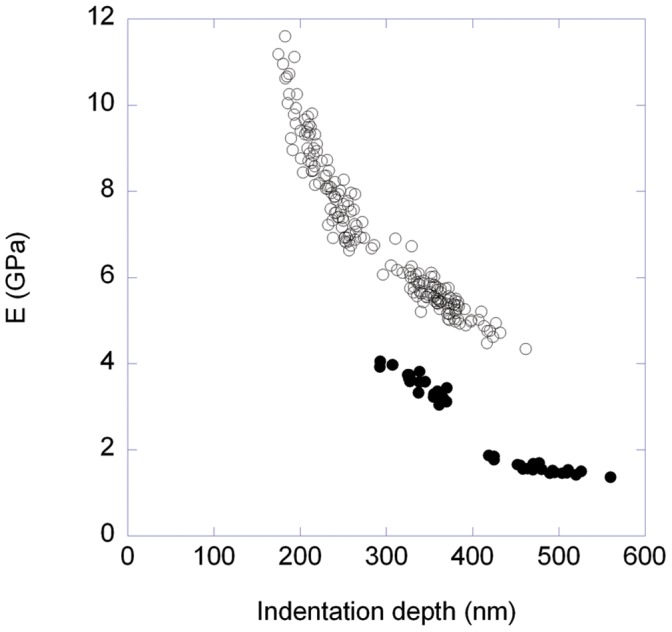
Dynamic Young’s modulus as a function of indentation depth. The Young’s moduli were extracted using the Oliver and Pharr method from force curves obtained at a velocity of 0.2 µm/s. Hair fibre with exposed cortical cells (open symbols, two different data sets), single cortical cell extracted from a porcupine quill (solid symbols, two different data sets).

### Atomic Force Microscopy (AFM)

All the measurements were carried out at an ambient relative humidity of 50%. For all the experiments we used a hemispherical, tungsten carbide-coated, cone-shaped probe (Team Nanotech) with a measured tip radius of 20 nm and a half-cone angle of 10°. This AFM probe had a spring constant of 600 N/m. All the experiments were carried out in air using a Bioscope Catalyst (Bruker) mounted on an IX71 inverted microscope (Olympus).

The samples were first imaged at a rate of 0.3 Hz in peak force tapping mode with a maximum load of 10 µN. In this imaging mode the instrument acquires a force curve at each pixel at a frequency of 1 kHz. This corresponded to tip velocities of either 400 or 600 µm/s. For each image, the instrument generated automatically an elastic map by fitting the retract curve at each pixel with the Derjaguin-Muller-Toporov (DMT) model [13] assuming a poisson’s ratio of 0.5 and a tip radius of 20 nm. We used these elastic maps for display and extracted a subset of curves that were analyzed using the Sneddon model for a conical indentor [14] and a poisson’s ratio of 0.5 to obtain an estimate of the dynamic Young’s modulus.

For each sample we also carried out nano-indentation with a tip velocity of 0.2 µm/s. The maximum load was 60, 90 or 120 µN. Each force curve was background corrected, and the point of first indentation was identified visually. The retract curves were fitted using the Oliver and Pharr method with a poisson’s ratio of 0.5 [15]. Briefly, for each retract curve we calculated the indentation depth *h_c_* and the stiffness *S*. The stiffness was obtained as the slope of a linear fit through the data between half the maximum load and the maximum load. Then these two values were combined in equation (1) [16], [17] to obtain the dynamic Young’s modulus *E*:
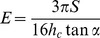
(1)


With α the half-cone angle of the tip.

**Figure 3:**
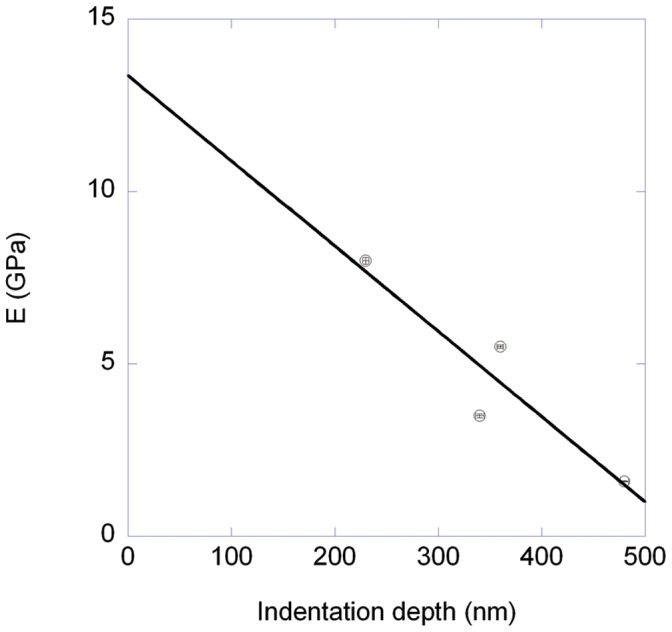
Average dynamic Young’s modulus as a function of average indentation depth. We used the four data sets shown in Figure 2 to plot the average dynamic Young’s modulus as a function of average indentation depth. The Young’s moduli were extracted using the Oliver and Pharr method from force curves obtained at a velocity of 0.2 µm/s. The linear regression through the data (solid line) gives an intercept of 13±3GPa.

**Table 1:** Summary of dynamic Young’s modulus measured on the hair and porcupine quill samples.

Sample	Velocity(µm/s)	Young’s modulus(GPa)	Depth(nm)	Maximum Load(µN)
Hair&	0.2	8±0.1 (n = 99)	230±3 (n = 99)	60
Hair&	0.2	5.5±0.05 (n = 98)	360±3 (n = 98)	90
Hair& (macrofibril)	400	87±3 (n = 36)	16.3±0.25 (n = 31)	10
Hair& (CMC)	400	120±3 (n = 27)	13.7±0.3 (n = 26)	10
Porcupine@	0.2	3.5±0.06 (n = 22)	340±4 (n = 22)	60
Porcupine@	0.2	1.6±0.02 (n = 22)	480±4 (n = 22)	120
Porcupine@ (macrofibril)	600	150±3 (n = 22)	16.2±0.3 (n = 22)	10
Porcupine@ (CMC)	600	228±8 (n = 20)	12±0.5 (n = 20)	10

& Hair fibre with exposed cortical cells.

@Single cortical cell extracted from a porcupine quill.

## Results and Discussion

### The Lateral Young’s Modulus of Cortical Cells is Depth Dependent

We used a sharp conical AFM tip to indent the surface of a cortical cell extracted from porcupine quills and of a human hair fibre with an exposed cortex. For indentation depths between 200 and 500 nm, we observed that the dynamic Young’s modulus at a velocity of 0.2 µm/s was inversely proportional to the indentation depth (Figure 2). There was a constant ∼2 GPa shift between the human hair fibre data (Figure 2, open symbols) and the porcupine quill data (Figure 2, solid symbols) for similar indentation depths. Based on these data, it was only possible to extract a dynamic Young’s modulus for the porcupine quill of 1.6±0.02 GPa. This is slightly lower than the tensile Young’s modulus, i.e. 2.5±0.5 GPa, measured on the cortex of porcupine quills of the same species at 65% relative humidity [18]. This is expected as we measured the lateral Young’s modulus rather than the longitudinal one as typically measured by tensile testing. One should also note that the published values of tensile Young’s modulus vary from 2 to 3.7 GPa for human hair and 1 to 3.5 GPa [19], [20] for porcupine quill [18]. This means that the 2 GPa shift between the human hair and porcupine quill data in Figure 2 can be accounted for by the natural variation in the samples.

In order to study the depth dependency of the dynamic Young’s modulus further, we looked at the average modulus as a function of the average indentation depth. We observed a linear trend with a dynamic Young’s modulus at zero indentation depth of 13±3 GPa (Figure 3). As a comparison, we measured the dynamic Young’s modulus of the macrofibrils using the peak-force mode. With this mode, we achieved indentation depths of less than 20 nm. The obtained dynamic Young’s modulus was 87±3 GPa for the hair sample and 150±3 GPa for the porcupine quill sample, at indentation velocities of 400 and 600 µm/s respectively (Table 1). To compare with the above-mentioned estimate, we need to take into account the large difference in velocities between the two kinds of measurements. It is well known that the dynamic elastic moduli of polymer materials increase as the logarithm of the probing velocity [21]. This leads to a corrected dynamic modulus at a probing velocity of 0.2 µm/s, of 11.5±0.4 GPa and 18.7±0.4 GPa, respectively. All together our data points to the fact that the Young’s modulus of the first 20 nm of a macrofibril is almost 10 times larger than the Young’s modulus of its core. Interestingly, a cell membrane complex (CMC) with a total thickness around 10 nm surrounds each macrofibril [6]. Until this study, there were no values available for the Young’s modulus of the CMC. Still, the exo-cuticle as a similar structure than the CMC and Parbhu et al. measured a Young’s modulus via nano-indentation of 20±8 GPa at a velocity of 0.1 µm/s [22]. In summary, we demonstrated that peak-force imaging combined with nano-indentation can reveal the skin-core architecture of keratin macrofibrils [6].

### Time-lapse Elastic Mapping of Macrofibrils’ Relaxation after Loading

**Figure 4:**
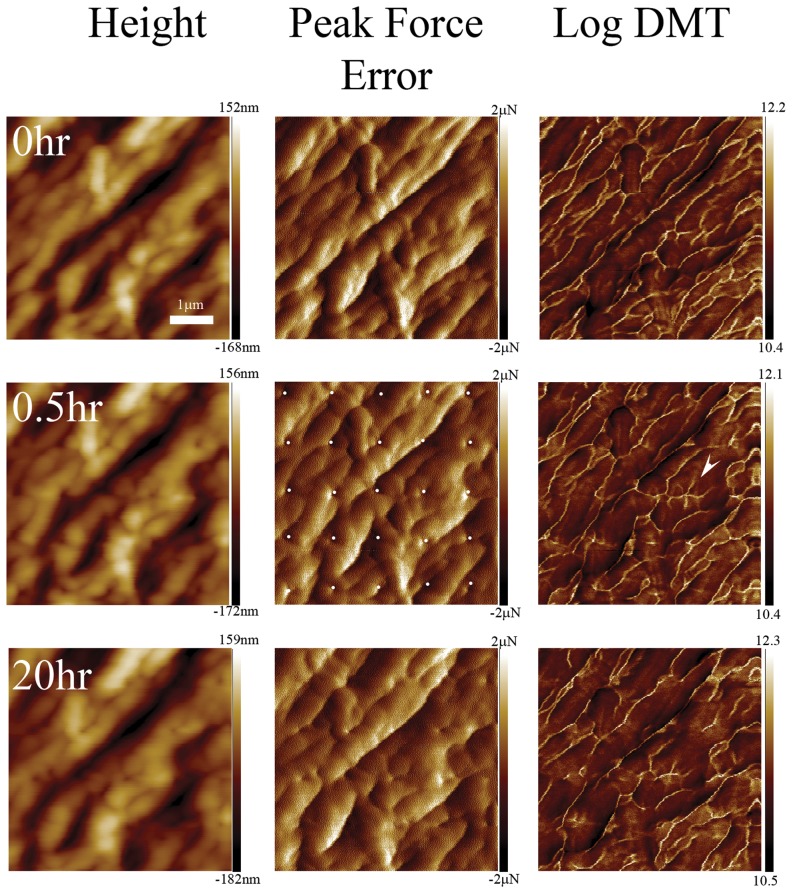
Time-lapse imaging of the surface of a porcupine quill’s cortical cell after indentation. AFM images obtained with peak force tapping mode of a cortical cell extracted from a porcupine quill, the scan size is 5 µm. The images were taken before, 30 min and 20 h after nano-indentation on a 5 by 5 grid with a maximum load of 120 µN. The indent positions are indicated by white dots. The elastic map channel (Log DMT) reveals that the cell membrane complex (CMC) between macrofibrils is stiffer than the macrofibril themselves. After indentation, the patterned formed by the boundaries was significantly disturbed and in one case (arrowhead) several lines of high stiffness radiated from an indent. After 20 h the entire elastic map reveals an increase in Young’s modulus compared to the reference map while the indents are still clearly visible.

**Figure 5:**
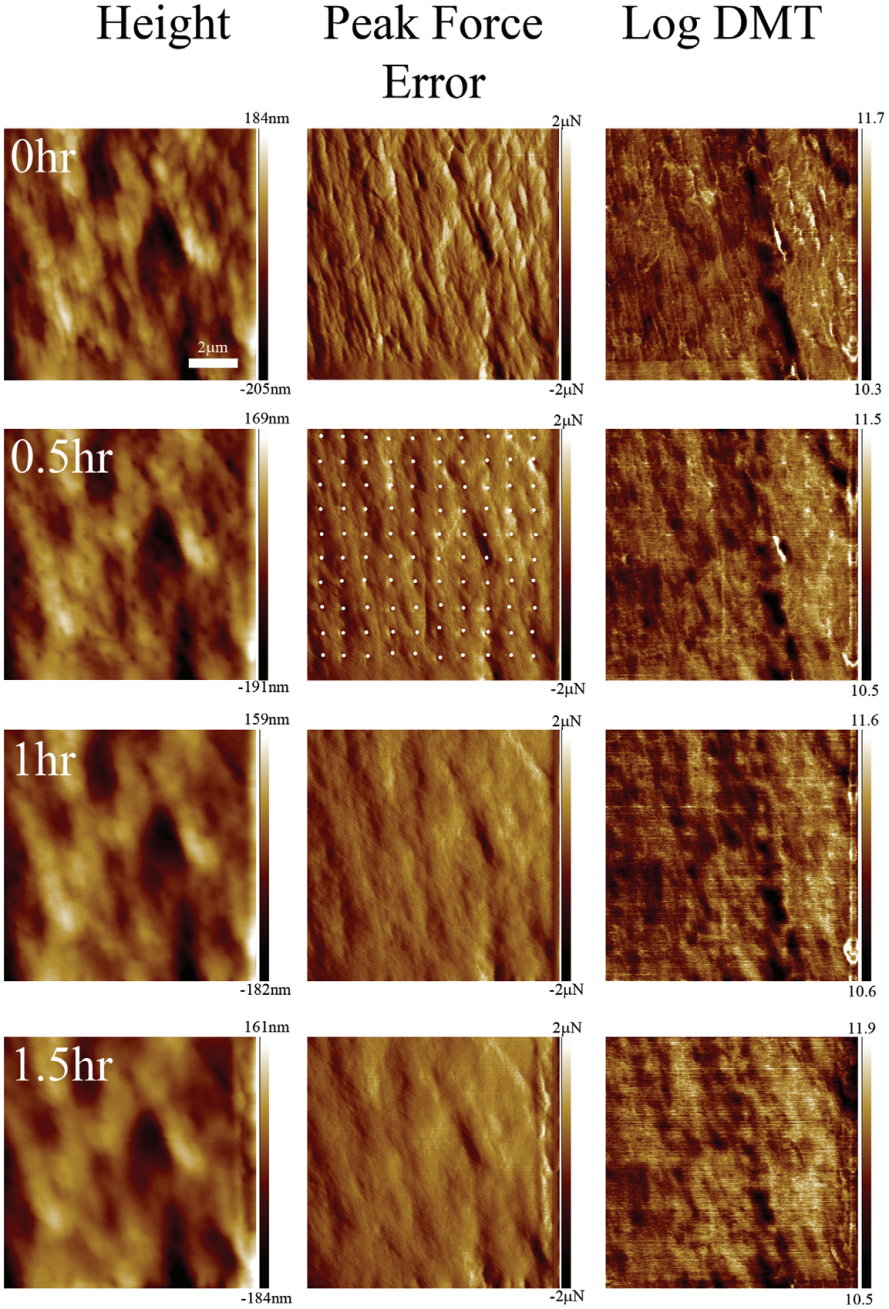
Time-lapse imaging of the surface of the cortex of a human hair after indentation. AFM images obtained with peak force tapping mode of the surface of a human hair fibre with exposed cortex, the scan size is 10 µm. The images were taken before, 30 min, 1 h and 1.5 h after nano-indentation on a 10 by 10 grid with a maximum load of 90 µN. The indent positions are indicated by white dots. After 1 h the indents have completely disappeared but the elastic map channel (Log DMT) reveals an increase in elastic modulus mainly at the indents’ sites.

**Figure 6:**
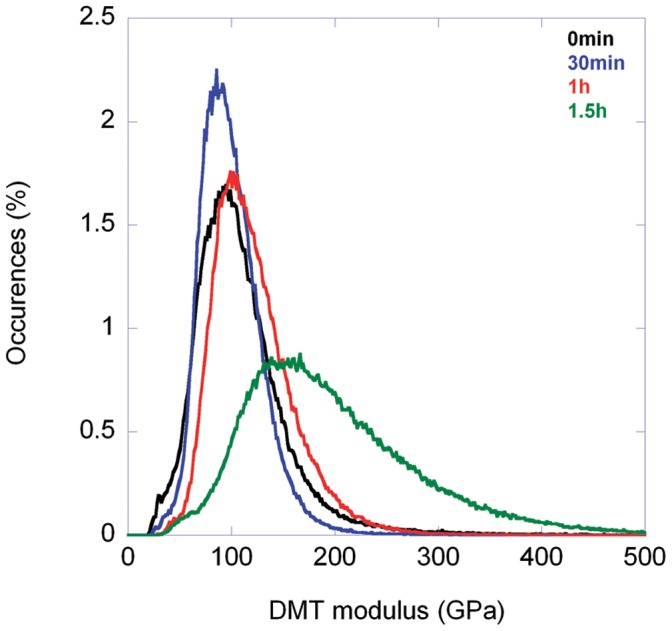
The cortex of a human hair stiffens after indentation. DMT modulus histograms of the elastic maps shown in Figure 5. The DMT modulus distributions follow Poisson-like statistics. The mean and the standard deviation of the distribution both increase with time indicating a stiffening of the sample.

In each relaxation experiment, a peak-force image was acquired at 10 µN force prior to nano-indentation at a velocity of 0.2 µm/s over a 5 by 5 or a 10 by 10 grid. Afterward, we followed the relaxation process in peak-force mode at a rate of one image per 30 min over up to a day. The elastic maps obtained before nano-indentation revealed the expected macrofibrillar texture (Figure 4) with the boundaries between the macrofibrils which are purely the CMC appearing 50% stiffer than the macrofibrils themselves as expected from the discussion in the previous section (Table 1). For the three maximum loads, 60, 90 and 120 µN, we always observed indents right after indentation. However the relaxation process was slightly different for the two samples. For the cortical cells extracted from porcupine quills the indents remained visible on the surface for 20 h (Figure 4). Still the depth of the indents decreased significantly over time as visualized by subtracting consecutive height images (data not shown). During the same period, the dynamic Young’s modulus of the area around the indents increased (Figure 4, 20 h panel). This global effect was accompanied by a local increase in dynamic Young’s modulus along lines centered on some of the indents (Figure 4, arrowhead). It is likely that these lines of higher modulus are due to the non-uniform strain field induced by the indenter. For the human hair fibres with exposed cortex, the indents disappeared completely between 30 min and 1.5 h (Figure 5). At the same time the elastic maps revealed an increase in dynamic Young’s modulus localized mainly at the indents’ sites (Figure 5). The DMT modulus histograms extracted from the elastic maps show both an increase in the average modulus as well as a shift of the entire distribution towards higher modulus (Figure 6).

Stress relaxation with a time constant of the order of hours has already been observed for a small deformation, 0.8% strain, of wool fibers [23]. However until this study there was no report of relaxation induced stiffening of keratin fibres or macrofibrils. What has been reported is that extension of a keratin fibre into the yield region leads to the unfolding of α-helices [9]. If one allows the fibre to relax in the presence of steam at constant length, the unfolded regions refold into either α-helical or stiff β-sheet rich domains [9]. Based on this finding and our present data, we propose that the increase in Young’s modulus around the indents over time is indicative of the unfolding of α-helices followed by a partial refolding into β-sheet domains upon relaxation. This assumes that the strain field applied by the indenter is above the yield strain of around 2–4%. For work hardening metals, the contact strain induced by a cone is roughly 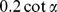
with α the half-cone angle [24]. Assuming that this formula still applies for keratin fibres, then it translates to an applied strain of 140% for the tip used in this study. This is far beyond the yield strain and may explain why we observed the α-helix to β-sheet transition at a relative humidity at which X-ray diffraction does not detect the presence of β-sheets in stretched keratin fibres [8]. Finally, it is important to mention that even so our data are consistent with the formation of β-sheet domains after indentation, it is still possible that some β-sheet formation occurs instantaneously as shown by recent steered molecular dynamics simulations of the human vimentin dimer and tetramer [25], [26].

### Conclusion

We combined nano-indentation with a sharp indenter and peak-force imaging to demonstrate that large strains applied at the nanoscale can induce strain hardening after relaxation in keratin macrofibrils. The molecular origin of this strain relaxation process is most likely the unfolding/refolding of the α-helical keratin molecules into β-sheets. Considering that chemists have now developed large arrays of helical polymers [27] it should be possible to design a fibrous composite that shows a similar nanoscale strain hardening behavior.
